# Empowering rare disease patients through patient education: the new BehçeTalk programme

**DOI:** 10.1186/s41927-022-00247-1

**Published:** 2022-02-28

**Authors:** D. Marinello, A. Del Bianco, A. Manzo, M. Mosca, R. Talarico

**Affiliations:** 1 Azienda Ospedaliero Universitaria Pisana, Rheumatology Unit, University of Pisa, Via Roma 67, 56126 Pisa, Italy; 2 Associazione S.I.M.B.A (Associazione Italiana Sindrome e Malattia di Behçet), Pontedera, Italy; 3 Rome, Italy

**Keywords:** Educational programme, Behçet’s disease, Patients' empowerment

## Abstract

**Background:**

Educating patients and caregivers on their disease can improve their knowledge and promote the active involvement in the therapeutic decision-making process. Naturally, patient education programmes are critically important in rare systemic autoimmune diseases, where relevant knowledge and expertise still remain scattered. Behçet’s disease (BD) represents a challenging rare condition, characterized by a variable spectrum of disease profile and a relapsing course.

**Results:**

Recently, BehçeTalk, an educational programme tailored for BD patients, families and caregivers with, was launched. BehçeTalk, entirely co-designed with BD patients, is offering educational on-line webinars on different aspects of the disease, as well support groups for patients and caregivers coordinated by a psychologist with specific expertise in BD.

**Conclusions:**

The therapeutical management of BD is often challenging and frequently includes off-label treatments. Considering the specificities of BD, providing a specific education on the disease to patients will lead to empower them in being part of the decision-making processes, in the self-management and in improving their quality of life.

**Supplementary Information:**

The online version contains supplementary material available at 10.1186/s41927-022-00247-1.

Sirs,

Patients’ education can be considered a milestone of the patients’ empowerment, in terms of knowledge and awareness on the disease and of rights and responsibility of the patient in the care process [1]. Educating patients and caregivers on their disease can improve their knowledge and promote the active involvement in the therapeutic decision-making process [2–4]. Naturally, patient education programmes are critically important in rare systemic autoimmune diseases, where relevant knowledge and expertise still remain scattered.

Behçet’s disease (BD) represents a challenging rare condition, characterized by a variable spectrum of disease profile and a relapsing course [5]. The therapeutical management of BD is often challenging and frequently includes off-label treatments. Considering the specificities of BD, providing a specific education on the disease to patients will lead to empower them in being part of the decision-making processes, in the self-management and in improving their quality of life.

Recently, BehçeTalk, an educational program tailored for patients, families and caregivers living with BD, was launched in Italy [6]; the initiative was promoted by the Behçet Clinic of Pisa together with the the Italian patients’ association for BD (SIMBA). BehçeTalk is offering educational on-line webinars on different aspects of the disease, as well support groups for patients and caregivers coordinated by a psychologist with specific expertise in BD.

BehçeTalk was entirely developed in co-design with BD patients, caregivers and patients’ representatives, by means of multisteps agreement on the the unmet nedds and relative topics to be covered. Involving patients and caregivers in the identification of each topic ensured that the programme is tailored to what patients consider important. Each webinar is presented in a standard format in which different experts in BD focus the talk on specific aspect(s) of the disease in the first section of the webinar; furthermore, a session questions and answers represents the second section of the talk. Some of the topics addressed in the programme include quality of life, workability, sexuality, self-management, adherence to treatment, pregnancy and family planning. Moreover, some of the major points to be discussed in the patients’ support groups are related to the ability to heal the “wound” resulting from the BD diagnosis, the perception and happiness in daily life, interpersonal and social relationships, while for BD caregivers being parents/partner/siblings of a BD patient represent the most frequent challenge.

The BehçetTalk educational program aims to create greater awareness of the disease and of its impact on the daily life of patients, partners, caregivers and family members. Being informed and acquiring a good level of awareness helps patients living better lives, but also helps doctors and patients in improving the management of the disease.

The more clearly BD is understood, the more likely BD patients and their caregivers can be comfortable with their disease and their care (Additional file 1).

## Supplementary Information

**Additional file 1**. Online the educational programme “BehçeTalk”.

## Data Availability

The material related to the educational program BehçeTalk are available on the website https://behcetclinic-pisa.it/en/behcet-talk-eng/.

## Abbreviations

BD: Behçet’s disease
S.I.M.B.A: Associazione Italiana Sindrome e Malattia di Behçet
