# Supplementary material for: Empowering rare disease patients through patient education: the new BehçeTalk programme
Source: BMC Rheumatol. 2022 Feb 28;6:17. doi: 10.1186/s41927-022-00247-1 (PMC8883708; doi:10.1186/s41927-022-00247-1)

**It is online the educational programme “BehçeTalk”**

BehçeTalk is an educational and support program for patients, family members and caregivers living with Behçet’s disease. The programme has been launched in the 2021 (behcetclinic-pisa.it/behçet-talk) by the Behçet Clinic of Pisa. The programme provides patients and clinicians different educational activities dedicated to Behçet’s disease, including both Public and Private Talks.

The Public Talks are held online and address important aspects of Behçet’s disease. The Public Talks are conducted by the most prominent specialists dealing with Behçet’s disease and cover key topics of the disease, such as quality of life, pregnancy, therapies and lifestyle, sexuality, impact of the disease on the work. They are available live (the schedule of the talks is available on the website, https://behcetclinic-pisa.it/behcet-talk-pubblici/) and the recordings are always published in the dedicated website and on the YouTube channel.

BehçeTalk also carries out in parallel, a series of group support meetings coordinated by a psychologist with experience in Behçet’s disease. The support groups are designed for patients and caregivers living with Behçet’s disease (including siblings, parents, husbands and wives of patients living with the disease). These meetings are dedicated to small groups of about 10 people each and take place on a monthly basis.

The Programme will be soon launched internationally with Talks held in English and published on the website.


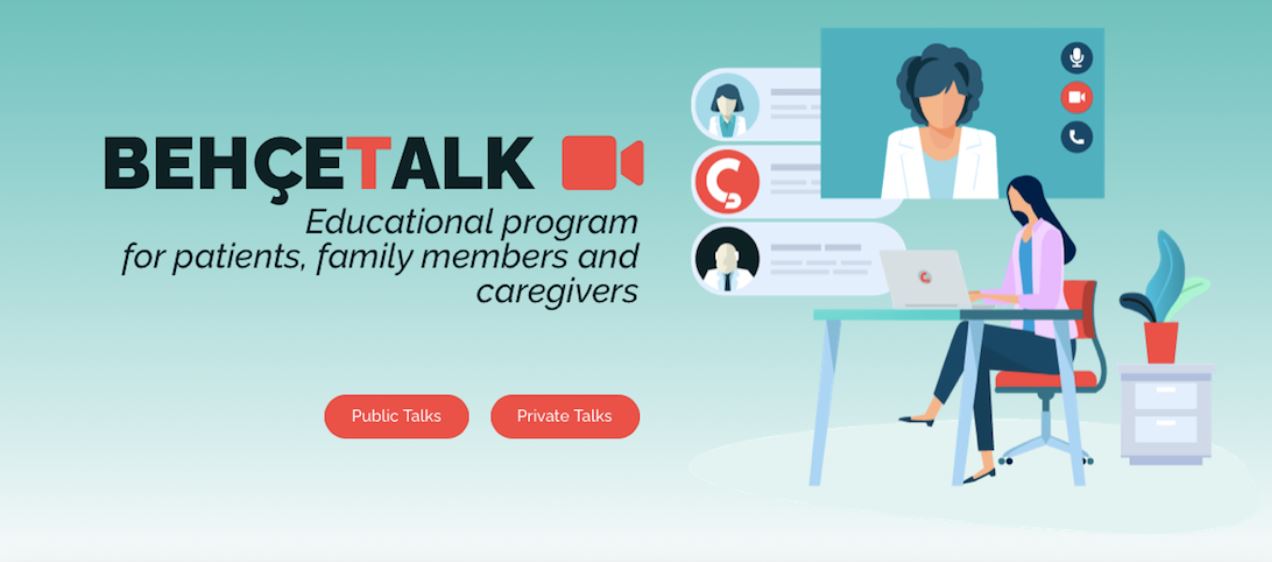

Supplement: Supplementary file 1 — Additional file 1. Online the educational programme “BehçeTalk”. [file 41927_2022_247_MOESM1_ESM.docx]
